# Mechanisms of pulmonary fibrosis: role of activated myofibroblasts and NADPH oxidase

**DOI:** 10.1186/1755-1536-5-S1-S23

**Published:** 2012-06-06

**Authors:** Victor J Thannickal

**Affiliations:** 1 Division of Pulmonary, Allergy and Critical Care Medicine, University of Alabama at Birmingham, 1530 3rd Avenue South, THT 422, Birmingham, AL 35294-0006, USA

## Abstract

A common feature of pathological fibrosis involving the lung and other organs is the persistent activation of myofibroblasts in injured tissues. Recent evidence supports the role of a member of the NADPH oxidase (NOX) gene family, NOX4, in myofibroblast differentiation, matrix synthesis and contractility. Additionally, NOX4 may contribute directly or indirectly to alveolar epithelial cell death, while myofibroblasts themselves acquire an apoptosis-resistant phenotype. Thus, NOX4 may be responsible for the cardinal features of progressive fibrosis - myofibroblast activation and epithelial cell dysrepair. Therapeutic targeting of NOX4 is likely to be effective in progressive cases of fibrosis involving multiple organs.

## Introduction

Fibrosis of mammalian tissues/organs is perhaps best understood as an evolutionarily conserved, adaptive tissue response to injury. In most pathological scenarios when tissue fibrosis is observed, there appears to be an associated impairment in regeneration of the adjacent airway/luminal epithelium or vascular endothelium. Natural selection appears to have favored the development of fibrosis at sites of wounding or injury, at the expense of complete restoration of tissue architecture and function. It makes teleological sense that the more immediate need for survival of the organism from bleeding and infection would take precedence over a temporary (often reversible), and often marginal, loss of organ functions. Indeed in multiple plant and animal species, formations of extracellular matrix (ECM) "scars" around traumatic, non-infectious and/or infectious injury are "normal" responses that serve to limit the invasion/spread of the pathogen at the site of injury/infection [1]. An illustrative example of this tissue response in humans is in the formation of a fibrotic scar around *Mycobacterium tuberculosis *bacilli, without which an estimated third of world's population infected with this infectious agent would not be expected to survive for very long [2]. Thus, fibrosis may be considered as part of innate host defense mechanisms against infection, or the perceived threat of infection (non-infectious injury). The enigma in many human highly lethal fibrotic disorders, in particular idiopathic pulmonary fibrosis (IPF), is the current, lack of understanding of: (1) the etiology of the apparent injury/infection, and (2) the progressive nature of the fibrotic process. Here, I address these issues with a greater focus on the second problem related to fibrosis progression and a potential role for NOX4 in this process.

## Etiological considerations in IPF

**Figure 1:**
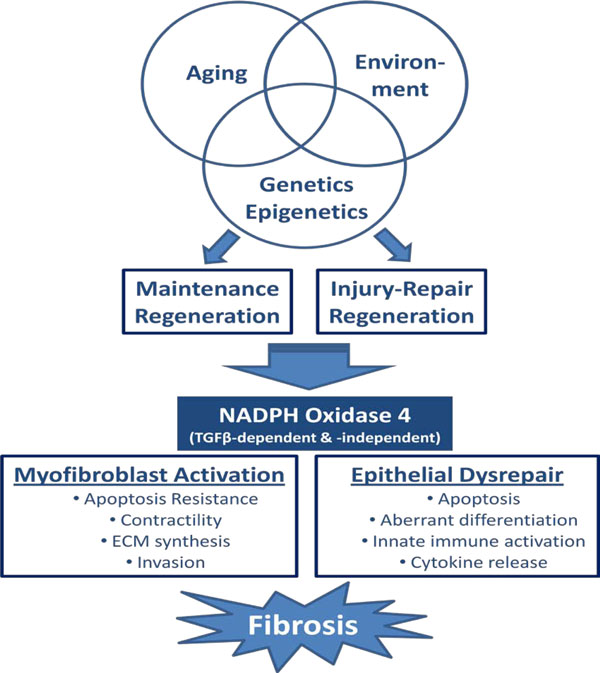
**Hypothetical model of a central role of NADPH oxidase-4 (NOX4) in pathological fibrosis**: Aging, environmental and genetic/epigenetic factors influence the persistent expression/activation of NADPH oxidase 4 (NOX4) in fibrotic tissues. This may be due to aberrations in injury repair responses or in maintenance regeneration, resulting in a failure to maintain cellular homeostasis in fibrotic tissues. Loss of tissue homeostasis is characterized by myofibroblast activation and epithelial cell dysrepair, with their attendant cellular phenotypes, that promotes pathological tissue fibrosis.

Pulmonary fibrosis results from a large number of known causes (e.g. organic and inorganic dust exposures); however, a specific etiological agent in IPF has not been identified [3]. A number of risk factors have been identified; these include inhalational exposures, including cigarette smoking, gastroesophageal reflux, diabetes mellitus, and advanced age. Given the natural course of the disease and the epidemiologcal data supporting the concept that IPF is an age-associated disease [4], one possibility is that this results from a failure of "maintenance regeneration" due to a combination of chronic, subclinical insults in a subset of genetically-susceptible, elderly patients (Figure 1). Indeed, telomerase mutations have been identified in familial IPF [5,6], and shortened telomeres appear to be a risk factor for sporadic IPF [7]. Fibrosis associated with "injury-provoked regeneration" which may be easier to recognize clinically due to the relative acuteness of disease onset and progression, as in diseases such as hypersensitivity pneumonitis.

## Fibrosis progression: persistence of tissue myofibroblasts

While a number of acute/subacute lung injuries that result in fibrosis are at least partially reversible, IPF is not. A key feature of these clinical syndromes in diverse organ systems is the persistence of tissue myofibroblasts in actively remodeling tissues. The local activation of tissue myofibroblasts are a highly conserved and stereotypic response to injury [8], and is fundamental to a broader biological principle in development, maintenance, and injury-repair responses of mammalian tissues/organs - perpetual and dynamic epithelial-mesenchymal-endothelial interactions.

In the repair response to injury, activation of local myofibroblast precursors occurs early and their disappearance is a hallmark of fibrosis resolution [9]. Mechanisms that lead to myofibroblast apoptosis in physiologic wound-repair, however, remain unclear; it has been postulated that cross-linking of a contracted ECM shields myofibroblasts from biomechanical stress and that loss of mechanical tension may induce myofibroblast apoptosis [8,10,11]. Similarly, the mechanisms of an apparent "apoptosis-resistant" myofibroblast phenotype in progressive fibrotic disorders, including IPF, are not well defined [12]. The presence, activation, and survival of myofibroblasts in fibrotic tissues explains several morphological, physiological, and clinical features, including the "fibrocontractive" nature of histopathological changes characterized by alveolar collapse, reduced lung compliance, restrictive physiology, and progression.

Our studies have demonstrated that, in addition to myofibroblast differentiation, the pro-fibrotic cytokine, transforming growth factor-β1 (TGF-β1), promotes myofibroblast differentiation [13]. TGF-β1 activates two pro-survival signaling pathways, focal adhesion kinase (FAK) and protein kinase B (PKB/AKT) by mechanisms that involve cell adhesion and release of soluble growth factors, respectively [13,14]; both pathways contribute combinatorially to myofibroblast survival [15]. Importantly, the administration of a protein kinase inhibitor that modulates the activities of these pro-survival pathways attenuates fibrosis in a model of bleomycin-induced lung fibrosis [16]. Further studies of the potential role of these pathways as a common mechanism of apoptosis resistance of myofibroblasts in IPF or in individualized patients requires further study.

More recent studies from our laboratory support the role of a member of the NADPH oxidase (NOX) family, NOX4, in myofibroblast differentiation/survival. NOX4 was identified as one of the most highly upregulated genes in transcriptomal (Affymetrix) analyses of human lung fibroblasts treated with TGF-β1 [17]. NOX4 activation mediates generation of hydrogen peroxide (H_2_O_2_), myofibroblast differentiation, contractility, and ECM production in response to TGF-β1, effects that also seen in human IPF-derived (myo)fibroblasts [17]. In human tissues of IPF patients, the expression of NOX4 is localized to myofibroblasts, both within fibroblastic foci and in remodeled blood vessels, as well as in epithelial cells associated aberrant bronchiolization [17]. Therapeutic targeting of this NOX isoform protects against fibrosis in two different animal models of injury-provoked pulmonary fibrosis [17]. Work by other investigators suggest that NOX4 may play a pro-fibrotic role by inducing apoptosis of lung epithelial cells [18]. Epithelial cell death may also be mediated indirectly by the paracrine secretion of H_2_O_2 _by activated myofibroblasts [19]. These observations, in addition to the purported role of NOX4 in vascular remodeling [20,21], suggest that NOX4 mediates effects on multiple cell types and tissue compartments that contribute to organ fibrosis.

## Conclusion

Progressive fibrotic disorders are associated with an apoptosis-resistant myofibroblast phenotype. The mechanisms that give rise to this myofibroblast phenotype may include the acquisition of pro-survival signaling pathways and expression/activation of NOX4. Paradoxically, activation of NOX4 and generation of extracellular H_2_O_2 _may promote the death of adjacent epithelial cells [19], setting up a feed-forward mechanism for fibrosis linked to impairments in epithelial regenerative capacity that would lead to progressive fibrosis. Targeting molecules and signaling pathways that promote survival of myofibroblasts represents a promising therapeutic strategy in clinical syndromes characterized by progressive of fibrosis, including IPF.

## Competing interests

The author declares that they have no competing interests.
